# European Code Against Cancer, 5th edition – outdoor and indoor air pollution and cancer

**DOI:** 10.1002/1878-0261.70184

**Published:** 2026-01-16

**Authors:** Gerard Hoek, Martie van Tongeren, Martin Röösli, Sylvia H. J. Jochems, Nadia Vilahur, Maria Albin, Isabelle Baldi, Quentin Crowley, Béatrice Fervers, Rüdiger Greinert, Dario Consonni, Ariadna Feliu, Hajo Zeeb, Joachim Schüz, Erica D'Souza, David Ritchie, Carolina Espina, Hans Kromhout

**Affiliations:** ^1^ Institute for Risk Assessment Sciences (IRAS) Utrecht University the Netherlands; ^2^ Centre for Occupational and Environmental Health, School of Health Sciences University of Manchester UK; ^3^ Swiss Tropical and Public Health Institute (Swiss TPH) Allschwil Switzerland; ^4^ University of Basel Switzerland; ^5^ European Agency for Safety and Health at Work (EU‐OSHA) Bilbao Spain; ^6^ Institute for Environmental Medicine Karolinska Institutet Stockholm Sweden; ^7^ Department of Laboratory Medicine Lund University Sweden; ^8^ Inserm U1219, Bordeaux Population Health Research Centre, EPICENE team Univ. Bordeaux France; ^9^ Service Santé Travail Environnement Pôle de Santé Publique, CHU de Bordeaux France; ^10^ Geology, School of Natural Sciences Trinity College Dublin Ireland; ^11^ Trinity Centre for the Environment Trinity College Dublin Ireland; ^12^ Department of Prevention Cancer Environment Centre Léon Bérard Lyon France; ^13^ Inserm U1296 “Radiation: Defense, Health and Environment” Lyon France; ^14^ Department of Molecular Cell Biology Skin Cancer Center Germany; ^15^ Occupational Health Unit Fondazione IRCCS Ca’ Granda Ospedale Maggiore Policlinico Milan Italy; ^16^ Environmental and Lifestyle Epidemiology Branch International Agency for Research on Cancer Lyon France; ^17^ Department of Prevention and Evaluation Leibniz ‐ Institute for Prevention Research and Epidemiology ‐ BIPS GmbH Bremen Germany

**Keywords:** air pollution, cancer prevention, combustion, european code against cancer, indoor, outdoor

## Abstract

Most European Union (EU) residents live in areas where outdoor air pollution levels exceed the 2021 World Health Organization (WHO) air quality guidelines for fine particles and nitrogen dioxide. Outdoor air pollution is classified as carcinogenic to humans, and both outdoor and indoor air contain established human carcinogens, including diesel exhaust particulates, benzo(a)pyrene [B(A)P] and benzene. The European Code Against Cancer, 5th edition (ECAC5), incorporates recommendations for individuals and policymakers aimed at reducing the cancer burden from both outdoor and indoor air pollution. A critical step is aligning EU air quality limit values with the more stringent 2021 WHO guidelines. This should be complemented by integrated policy measures, including stricter regulation of combustion emissions, promotion of active and environmentally friendly transportation, incentives for cleaner energy sources for heating and cooking, and harmonization with broader EU climate initiatives. At the individual level, emissions and exposure may be reduced by limiting car use, avoiding second‐hand smoke, and refraining from burning wood or coal indoors or outdoors. Further exposure reduction may be achieved by limiting walking or cycling along heavily trafficked routes.

## Graphical abstract

The 5th edition of the European Code Against Cancer (ECAC5) has 14 recommendations on cancer prevention. Here, we present updates on cancer prevention related to air pollution, positioned as recommendation number 11. It recommends: (1) using public transportation and walking or cycling instead of using a car; (2) choosing low‐traffic routes when walking, cycling or exercising; (3) keeping your home free of smoke by not burning materials, such as coal or wood; and (4) supporting policies that improve air quality.

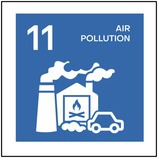

**Abbreviations**
[B(A)P]: benzo(a)pyrene
COPD: Chronic Obstructive Pulmonary Disease
EBCP: Europe's Beating Cancer Plan
ECAC: European Code Against Cancer
ECAC4: European Code Against Cancer, 4th edition
ECAC5: European Code Against Cancer, 5th edition
EU: European Union
IARC: International Agency for Research on Cancer
NO_2_
: Nitrogen dioxide
PAHs: Polycyclic Aromatic Hydrocarbons
PCBs: Polychlorinated biphenyls
PM_10_
: Particulate matter with a diameter less than 10 micrometres
PM_2.5_
: Particulate matter with a diameter less than 2.5 micrometres
WHO: World Health Organization

## 1 Introduction

On a global and European scale, air pollution is the largest environmental risk factor affecting human health [1]. Both outdoor and indoor air pollution affect human health. Air pollution is a complex and variable mixture of pollutants and has been linked to a wide range of health outcomes, including mortality and morbidity from cardiometabolic, respiratory, neurological causes and cancer [2]. In 2013, the International Agency for Research on Cancer (IARC/WHO) classified outdoor air pollution, and more specifically particulate matter, as carcinogenic to humans because of human, animal and mechanistic evidence linking air pollution to lung cancer [3, 4]. Major outdoor air pollutants related to lung cancer include particulate matter less than 2.5 micrometre (PM_2.5_) and nitrogen dioxide (NO_2_) [5, 6]. The classification of outdoor air pollution as a human carcinogen is consistent with the presence of individual compounds, previously designated as human carcinogens, including benzo(a)pyrene (BaP), cadmium, dioxins, formaldehyde and benzene, and major combustion sources, specifically diesel engine exhaust and automotive gasoline [7, 8, 9, 10, 11].

Most people in the European Union (EU) live in places where the outdoor air pollution levels exceed the latest (2021) World Health Organization (WHO) guidelines on the maximum levels of major pollutants in the air [12, 13, 14]. Because of long‐range transport of air pollution, large areas experience elevated concentrations. Large variations of outdoor air pollution are observed across the EU, with the lowest levels typically observed in Northern Europe [13] while the highest concentrations of PM_2.5_ occur in south‐eastern Europe. The highest concentrations of NO_2_ occur in urban areas across Europe [13].

A large variety of sources influence outdoor air pollution, including motorized traffic, industry, agriculture and residential burning of fossil fuels [15, 16]. Hence, policies addressing different economic sectors are needed to reduce outdoor air pollution levels.

Indoor air quality is influenced by outdoor air and indoor sources of pollution, including second‐hand smoke from tobacco smoking, cooking and burning of fossil fuels, such as coal, wood, natural gas and candles. The IARC Monograph on household solid fuel use classified household coal use as carcinogenic (Group 1) and biomass use (e.g., wood) as probably carcinogenic (Group 2A) [17]. Burning coal, wood and other materials (e.g., candles and incense) releases chemicals, such as polycyclic aromatic hydrocarbons (PAHs), fine particulate matter, formaldehyde and benzene, which have been classified as carcinogenic. Exposure to second‐hand tobacco smoke is also a cause of cancer [18].

The European Code Against Cancer (ECAC) is an initiative of the European Commission designed to provide clear, evidence‐based recommendations for cancer prevention, accessible to the general public. The 5th edition was coordinated by IARC as part of the World Code Against Cancer Framework, launched in 2022 to support the development of region‐specific Codes tailored to distinct epidemiological and socio‐economic contexts [19]. A specific methodology has been constructed for use in the development of any Regional Code, including ECAC5 as described in Espina et al. [20].

**Fig. 1:**
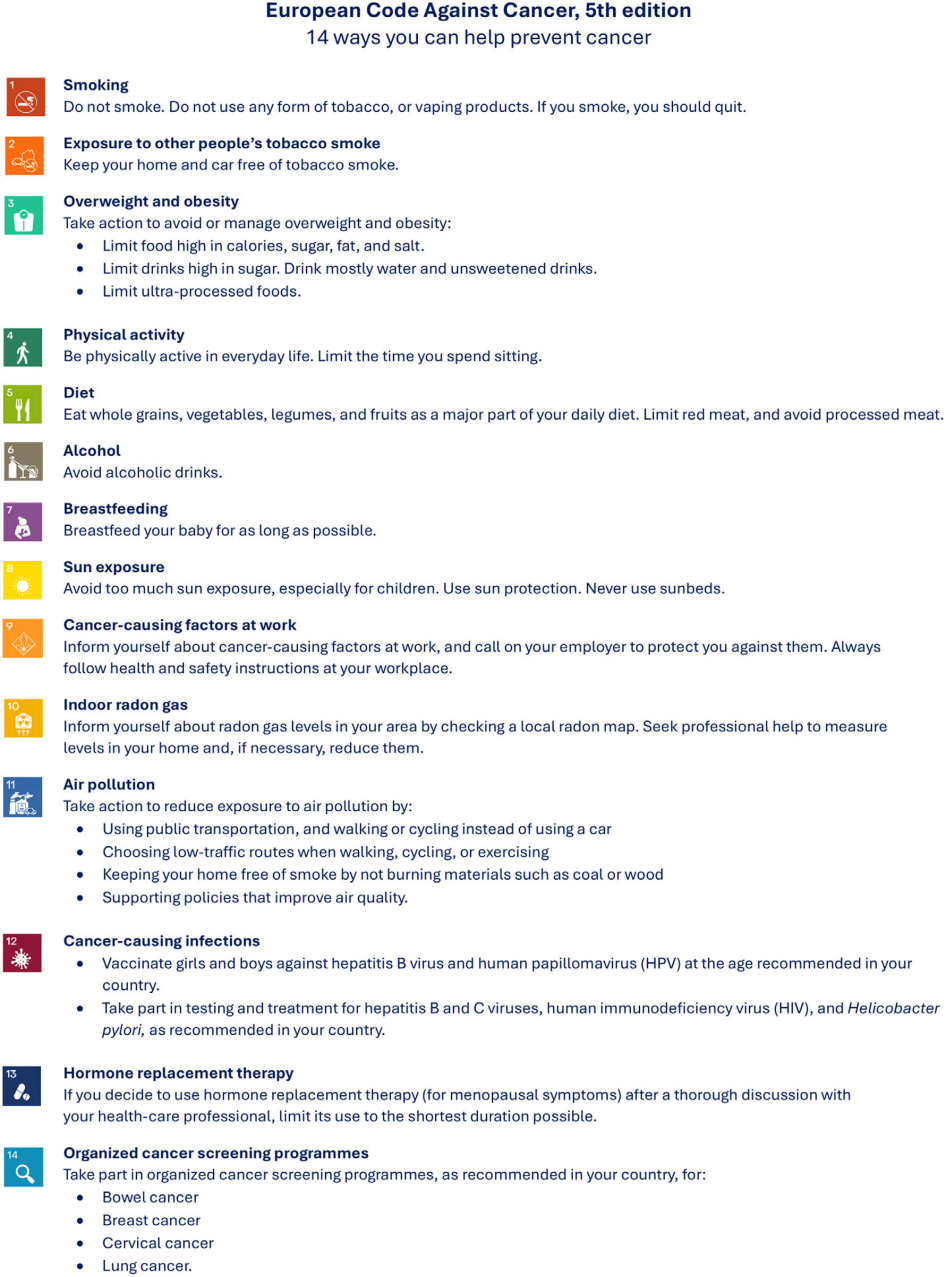
European Code Against Cancer, 5th edition: recommendations for individuals. The 14 recommendations of the European Code Against Cancer, 5th edition (ECAC5) adopted by the Scientific Committee of the ECAC5 project. © 2026 International Agency for Research on Cancer / WHO. Used with permission.

ECAC5 builds on the 4th edition (ECAC4) [21], also coordinated by IARC, by integrating the latest scientific evidence in cancer prevention. For the first time, ECAC5 is aimed not only at individuals (Fig. 1) but also at policymakers, including 14 complementary recommendations on a population level that may reinforce the 14 recommendations for individuals (Annex S1). Further details about the ECAC5 project are provided in Espina et al. [22].

This paper presents the scientific rationale for the recommendations targeted towards individuals and policymakers to reduce the cancer burden related to outdoor and indoor air pollution exposures.

## 2 Recommendation for individuals

### 2.1 Scientific justification for inclusion of the recommendation in ECAC5

The ECAC5 experts working group on Environment and Occupational Determinants reviewed the most recent scientific literature on air pollution, following the IARC methodology [20], and decided to include a new recommendation in ECAC5:

Take action to reduce exposure to air pollution by:

- Using public transportation, walking or cycling instead of using a car
- Choosing low‐traffic routes when walking, cycling or exercising
- Keeping your home free of smoke by not burning materials, such as coal or wood
- Supporting policies that improve air quality

As a principle, to modify, adapt or introduce a new recommendation in the ECAC, the current scientific body of evidence should be classified as ‘sufficient’ to demonstrate that adopting the recommendation would lead to reducing an individual's risk of developing cancer. In addition, contextual factors, such as equity, suitability, actionability and acceptability of the proposed recommendation in the context of the EU have been addressed by the working group.

### 2.2 Evidence on the association between exposure to air pollution and cancer

#### 2.2.1 Outdoor air pollution

Since the classification of outdoor air pollution as a Group 1 human carcinogen in 2013 [4], a sizable number of cohort studies have been published addressing associations between long‐term exposure to outdoor air pollution and cancer incidence or mortality. Reviews by WHO have summarized these studies focusing on lung cancer incidence or mortality [23, 24, 25, 26]. Based on a systematic review of the latest scientific evidence on health effects of human air pollution exposure, WHO has updated its air quality guidelines in 2021, based on evidence of mortality, including lung cancer mortality from NO_2_, PM_2.5_ and PM_10_. The evidence for lung cancer mortality for PM_2.5_ was rated at high certainty and moderate for PM_10_ [23]. A large review rated the evidence for traffic‐related air pollution and lung cancer as high certainty with a GRADE‐type approach and moderate with a narrative review [27]. Outdoor air contains several individual pollutants that are established carcinogens. Benzene is a known human carcinogen causing acute myeloid leukaemia in adults and possibly other neoplasms, evaluated by IARC in 2018 [10]. BaP, cadmium, dioxins and PCBs and diesel engine exhaust are further established carcinogens. Recently, gasoline automotive emissions were designated as an established carcinogen [11].

In the most recent systematic review commissioned by WHO for NO_2_, a summary relative risk of 1.07 (95% CI 1.04, 1.10) per 10 μg·m^−3^ was found for lung cancer mortality, based on 20 studies, including more than 100 million study participants [26]. In the most recent systematic review for PM_2.5_, a summary relative risk of 1.09 (1.05–1.13) per 10 μg·m^−3^ was found for lung cancer mortality, based on 26 studies [25]. Outdoor air pollution may also be associated with poorer cancer survival, though further research is needed [28].

There is increasing evidence that outdoor air pollution is associated with other cancers, including bladder, breast, colorectal, kidney and brain cancers [29, 30, 31, 32, 33]. The evidence for these cancers is, however, still less certain compared with lung cancer, partly because of a more limited number of studies. The finding of associations with cancers beyond the respiratory system is consistent with findings of noncancer health effects in multiple organs, including metabolic and neurological diseases related to systemic oxidative stress and inflammation and uptake of pollutants in blood [2].

Cohort studies that have evaluated the shape of the exposure response function have documented that there is no safe level below which long‐term air pollution has no effect [34]. The implication of a continuous exposure response function is that policies targeting any reduction of air pollution will lower the cancer burden.

**Fig. 2:**
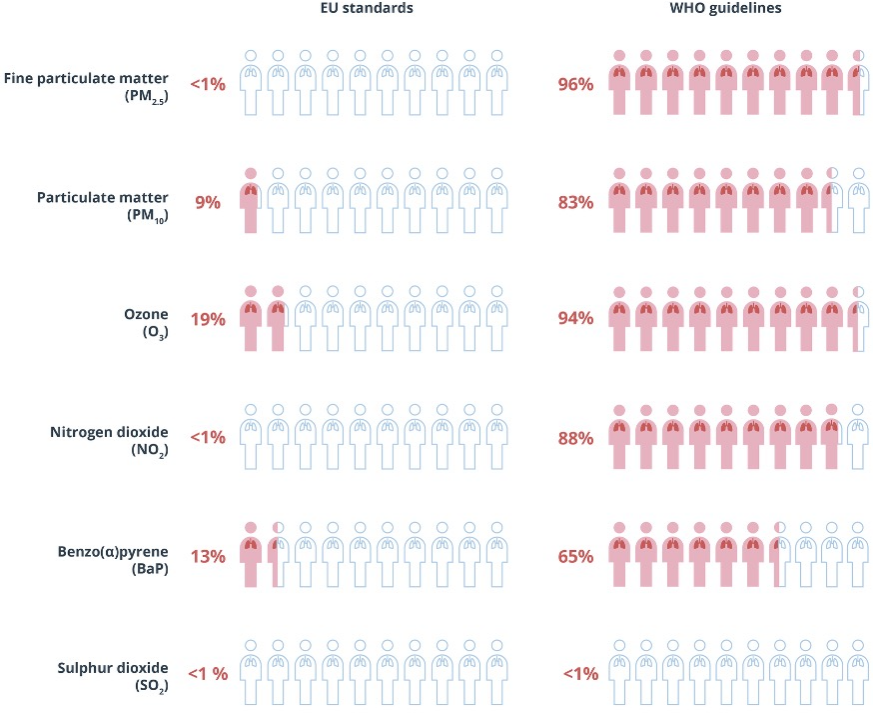
Share of the European Union (EU) urban population exposed to air pollutant concentrations above certain EU standards (valid in 2022) and World Health Organization (WHO) guidelines in 2022. Source EEA (https://www.eea.europa.eu/publications/europes‐air‐quality‐status‐2024). Standards and guidelines are listed in Table 1. Outdoor air pollution concentration levels for annual average exposure of fine particle matter (PM_2.5_), particle matter (PM_10_), ozone (O_3_), nitrogen dioxide (NO_2_), benzo(a)pyrene [B(A)P] and sulfur dioxide (SO_2_).

Although outdoor air pollution concentration levels have decreased substantially in the past decade [13, 14, 35], most areas in the EU still exceed the WHO guideline values for annual average exposures of PM_2.5_ and NO_2_ of 5 and 10 μg·m^−3^ (Fig. 2). Information about current and historic exposure levels can be found at the website of the European Environment Agency [14, 36]. Table 1 lists the WHO guideline values and the current and new EU limit values. The new EU limit values valid in 2030 for PM_2.5_ and NO_2_ are exceeded in a large number of countries and EU cities [36].

**Table 1:** Air quality limit values of the European Union (EU) and World Health Organization (WHO) guideline values for annual average outdoor concentrations of PM2.5 and NO_2_ (μg/m^3^) [36].

	EU current	EU 2030	WHO guideline
**PM**_**2.5**_	25	10	5
**NO**_**2**_	40	20	10

In 2023, 28 000 cancer deaths in Europe were estimated to be caused by particulate matter in the outdoor air [37]. Based on the recent systematic reviews and meta‐analyses [25, 26], it is possible to estimate the decrease in population attributable fraction of lung cancer related to specific exposure reductions. We translated the difference in concentration between the current and new EU standards, and the WHO guideline values, into population attributable fractions using standard health impact assessment methods [38]. Table 2 documents that the population attributable fraction for lung cancer can be reduced by more than 10% by reducing air pollution levels from the current EU limit values of 25 and 40 μg·m^−3^ for PM_2.5_ and NO_2_ to the levels proposed in the WHO guidelines. Reductions of about 5% in lung cancer can be achieved by reducing the PM_2.5_ and NO_2_ from the new EU limit values to the WHO guidelines.

**Table 2:** Decrease in population attributable fraction of lung cancer mortality related to annual average outdoor concentrations of PM2.5 and NO_2_ (μg/m^3^) at current and new European Union (EU) limit values compared with the World Health Organization (WHO) air quality guideline. Calculated using the relative risks (RR) from Kasdagli et al. and Orellano et al. [25, 26].

	EU current	EU 2030
**PM**_**2.5**_	0.16	0.04
**NO**_**2**_	0.13	0.07

In the EU, associations between air pollution exposure and socio‐economic position (income and education) are inconsistent and differ by geographical scale evaluated [39]. At the European scale, low socio‐economic position has been associated with high exposure. At the national scale, associations between socio‐economic position and exposure are variable. In cities, even more diverse findings have been obtained, for example, in London associations differed between central and more suburban areas, or in multiple Italian cities, people with a high socio‐economic position tend to have higher exposures because only wealthier people can afford to live in the more polluted city centres [39]. In a study in six medium‐sized European cities, lower income was associated with higher air pollution and green in some cities and lower air pollution and green in other cities [40].

#### 2.2.2 Indoor air pollution

Indoor air quality can be affected by outdoor air pollution that infiltrates buildings, as well as by indoor sources. Indoor sources of air pollution include the burning of materials (e.g., tobacco, fuels for heating and cooking, candles and incense), which results in the emission of particulate matter, gases (carbon monoxide and nitrogen oxides) and agents, such as PAHs. Activities such as home improvement (e.g., painting) and cleaning can result in the emission of volatile organic compounds (VOCs) into the indoor environment. Additionally, hobbies, pets and factors, such as dampness can lead to the presence of other chemicals, pesticides and microorganisms (bacteria and moulds) in the air. The result is often a complex and highly variable level and composition of indoor air pollutants, depending on factors, such as activities, sources, building type and ventilation [41, 42].

Compared with outdoor air pollution, there is much less information available on the carcinogenic risk of indoor air pollution. However, second‐hand tobacco smoke and the household use of coal have been classified as Group 1 carcinogens, while the indoor burning of biomass fuel has been classified as probably carcinogenic (Group 2A) by the International Agency for Research on Cancer (IARC) [17]. In compliance with the IARC methodology, a systematic review was produced to update the evidence on wood burning in a previous project to develop the Latin America and Caribbean Code Against Cancer, 1st edition (LAC Code) [43]. The review reported sufficient evidence for a link between biomass smoke exposure in homes, especially with inadequate ventilation, and lung and oesophageal cancers. Limited information was available regarding gastric and colorectal cancer, with indications of an increased risk [43]. Based on the results of the review, indoor burning of biomass fuel was included in the respective recommendation for the LAC Code. Most evidence in this review comes from studies carried out in Asia and Africa, raising questions on the applicability in European settings. Consistent with the IARC methodology, this evidence remains relevant for the ECAC5 assessment of the association with cancer.

Despite differences in the composition and levels of agents between indoor and outdoor air, there are also similarities in terms of particulate matter and gaseous components. Furthermore, outdoor air pollutants contribute to indoor air quality, while domestic sources, such as wood burning are an important component of outdoor pollution. Additionally, indoor pollution can include chemical agents, such as PAHs, formaldehyde, benzene and tobacco smoke, which are proven carcinogens.

Although indoor air pollution is not itself classified as a Group 1 carcinogen, it can be deduced with reasonable confidence that exposure to indoor air pollution is a risk factor for lung cancer based on:

- The presence of Group 1 carcinogens such as second‐hand smoke and emissions from coal burning, PAHs, formaldehyde and benzene;
- The similarities in sources and composition of the indoor and outdoor air pollution; and
- The results from the literature review carried out by the Latin America and Caribbean Code Against Cancer on the link between exposure to smoke from biomass fuel burning in the home and lung and oesophageal cancers.

### 2.3 Presentation of the recommendation

Many measures have been identified by a WHO working group that individuals can use to reduce their personal exposure [44]. We further assessed recommendations made by the US EPA for individuals to reduce exposure [45]. We have evaluated measures and selected those that are effective, feasible and without high costs for the individual. We further selected interventions that affect long‐term exposure to air pollution. Interventions that are designed to reduce acute health effects related to short‐term exposure were not considered.

*Use public transportation; walk or cycle instead of using a car.*

Using transportation other than gasoline‐ or diesel‐powered cars and motorcycles can contribute to reducing air pollution, especially in urban areas [46]. The background of this recommendation is the large contribution of motor vehicle emissions to urban air pollution [27]. Major roads are pollution hotspots in cities related to fossil fuel (gasoline and diesel)‐powered motor vehicles and nontailpipe emissions from brake, tyre and road wear. Emissions are lower per person‐kilometre for public transportation. Active travel (cycling or walking) or using public transportation reduces air pollution and has the added benefit of increasing physical activity [47]. By using public transport and active travel, individuals can limit their contribution to outdoor air pollution concentrations, which can be effective if large groups make this choice. This recommendation is easier to implement in cities than in more rural areas with less frequent public transport systems and larger distances. Car‐sharing is an alternative to reduce emissions.

Active travel and public transport do not necessarily lower individual exposure to air pollution; however, the benefits of active travel strongly outweigh the risks related to increased inhaled air pollution dose [48, 49, 50]. A review of studies comparing air pollution exposures in passive and active travel found that the differences between modes varied across studies [51].

*Choose low‐traffic routes when walking, cycling or exercising.*

Many studies have documented that route choice in urban areas significantly affects air pollution exposure during commuting. Levels of ultrafine particles, diesel engine exhaust, benzene and NO_2_ have been found to increase up to twofold on major roads compared with nearby minor roads. Route choice has been studied, especially in cyclists and pedestrians [52, 53, 54, 55, 56, 57, 58]. Concentrations decrease rapidly with increasing distance from a major road; for example, 50 metres from an inner‐city major road, concentrations are no longer higher than the urban background. In a study in Utrecht, ultrafine particle exposure on the minor road route was only 30% of that of the major road route from the city centre to the university area [52]. For 1‐h commutes, route choice may lower 24‐h average exposure by about 5%, a modest but non‐negligible decrease. For longer commutes, larger reductions are achieved. The recommendation is especially relevant for cyclists and pedestrians because they have more choice to select routes compared with car drivers. Furthermore, we do not want to encourage car drivers to use smaller (residential) roads. Choosing low‐traffic routes not only reduces exposure to air pollution but also reduces risks of accidents and exposure to traffic‐related noise.

*Keeping your home free from smoke by not burning materials, such as coal or wood.*

Avoid second‐hand smoke from tobacco and burning coal or wood. Indoor air pollution originates partly from polluted outdoor air, so it is also important to avoid burning materials outside. While tobacco smoking is the largest source of indoor air pollution, this is addressed in the specific ECAC5 recommendation on exposure to other peoples' tobacco smoke [59].

The statement on indoor air pollution in the ECAC5 air pollution recommendation is effective for reducing exposure to both indoor and outdoor air pollution. Moreover, while biomass is often promoted as a renewable and potentially low‐carbon energy source, its combustion for heat or power generation emits a range of harmful air pollutants, including particulate matter, NO_2_, PAH and others, and could have adverse air quality impacts. Woodsmoke has become a large contributor to particle emissions in European countries, partly because of decreasing emissions from other (traffic and industrial) sources [60, 61]. Although gas cooking is an important source of indoor nitrogen dioxide and particle pollution, there is no strong evidence of carcinogenicity. Cooking fumes from high‐temperature frying are classified as probably carcinogenic (2A), but we consider this more unlikely in the home, and evidence is mainly based on occupational exposures of cooks. Natural gas and electricity are the dominant sources for heating in European homes. We do not provide specific recommendations for these sources as there is no strong evidence that they affect indoor air quality. Reducing gas and oil combustion in the home is important for other noncancer health effects and reduction of greenhouse gases.

Evidence shows that low‐income households face higher exposure to indoor air pollution and are more vulnerable to its effects [62]. Additionally, people of lower socio‐economic status may demonstrate behaviours or conditions that worsen exposure, such as tobacco smoking or living in overcrowded dwellings. These inequalities could be reduced by improving indoor air quality.

*Support policies to improve air quality.*

Reducing outdoor air pollution is primarily the responsibility of local, national and EU authorities, major industrial facilities, car manufacturers and the agriculture sector that emit large amounts of air pollutants. Because there are many sources that affect air quality and major air pollutants remain airborne for a long period, everyone is exposed to air pollution. Individuals can only influence their own exposure to a limited extent. Therefore, promoting action from authorities is an effective strategy. There is a long history of organized citizens stimulating authorities to take action to improve air quality. This ranges from local issues, for example, around large polluting point sources, to organizations influencing EU policies. An example of the latter is the Health and Environment Alliance, which is very active in influencing the new EU air quality directive for outdoor air pollution and climate change policies. In recent years, citizen involvement has included actions linked to citizen science [63]. An example is the Italian NO_2_, NO Grazie project, in which citizens measured NO_2_ with passive samplers in collaboration with the University of Milan [64]. The project was conducted in the city of Milan in response to the Diesel‐Gate project and aimed at developing maps of NO_2_ based on measurements instead of modelling of potentially underestimated emissions. The results of the project contributed to a diesel ban in the city of Milan. In the Netherlands, a citizen science project documented health effects related to neighbourhood‐level wood burning [65]. Citizens use the results of this study to promote stricter regulation of wood burning. Citizens should also advocate for policymakers to implement policies that enhance indoor air quality in alignment with broader air pollution guidelines. This should be accompanied by targeted awareness campaigns to educate individuals on the risks associated with indoor burning of materials for heating, cooking and recreational purposes. Additionally, incentives should be provided to promote alternative methods, such as heat pumps or solar energy.

### 2.4 Feasibility, suitability, actionability and acceptability considerations

Each statement of the recommendation is feasible for individuals to implement, without high costs. The feasibility of the choice of active travel or public transport depends on the setting, for example, the availability of a proper infrastructure to safely cycle and the density and frequency of the public transport network. In general, in EU cities, these infrastructures have become increasingly available. Avoiding burning wood is relatively easy if the purpose of burning is for pleasure, as is often the case in urban areas. If wood (or coal) is used as a cheaper option to heat the home, avoidance is more challenging, particularly for low‐income families, but targeted policies can change this situation. Choosing low‐traffic density routes in urban areas is a simple intervention that typically does not lead to substantially longer travel times in urban areas. The choice has additional benefits, including lower accident risks and reduced exposure to traffic‐related noise. To successfully demand action from authorities typically requires significant levels of expertise and considerable time and effort from various stakeholders.

All recommendations, including supporting policies to improve outdoor air quality, do not require specific resources for any individual to implement. For indoor air quality, this is mainly based on behavioural change, which implies no significant cost. Overall, *costs* for the individual are considered to be negligible.

### 2.5 Co‐benefits for prevention of noncommunicable diseases other than cancer with similar risk factors and opportunities for health promotion

Outdoor air pollution is associated with several other health effects in addition to cancer, especially chronic and acute respiratory diseases asthma, chronic obstructive pulmonary disease (COPD) and cardiovascular diseases, which can result in premature death [1, 2, 12]. Evidence is increasing that air pollution is associated with adverse birth outcomes, diabetes and neurological diseases. Household air pollution has been linked with acute respiratory diseases in children and adults. Exposure to household air pollution can cause noncommunicable diseases, including stroke, ischaemic heart disease, COPD. Therefore, taking steps to decrease exposure to outdoor and indoor air pollution has several additional benefits for health. For a quantification of the benefits of air pollution reductions, we refer to the health impact assessment in 1000 European cities [16, 66].

Many policies related to air pollution reduction, including the use of public transportation or active travelling, have co‐benefits by reducing noise exposure and increase physical activity. These measures yield thus to an improvement in the health of the population; for example, noise is associated with numerous cardiometabolic and mental diseases [67, 68]. Policies to reduce intensive livestock agricultural emissions involving reductions of number of animals may have further benefits in reducing meat consumption [69].

## 3 Recommendations for policymakers

Air pollution is affected by many sources, at different spatial scales. Sources can be local, national or even international. Key sectors contributing to emissions of particles and NO_2_ include transport (road, shipping), industry, energy production (commercial and residential) and agriculture [70]. Agriculture is the dominant source of ammonia, which, through atmospheric reactions with nitrogen oxides, results in secondary fine particles. Policies need to address this complexity and account for the full range of air pollution sources, which either directly or indirectly are a contributing factor to human carcinogens. Table 3 shows the recommendations for policymakers on indoor and outdoor air pollution to reduce cancer risk, which are supported in part by Europe's Beating Cancer Plan [71] and the WHO NCD best buys [72].

**Table 3:** European Code Against Cancer, 5th edition: recommendations for policymakers on indoor and outdoor air pollution.

Indoor and outdoor air pollution
• Fully align EU air quality limit values with WHO global air quality guidelines for outdoor air pollution without delay. At the local and national levels, develop and implement plans to ensure that levels of all air pollutants comply with the WHO guidelines.
• Ensure further reductions in industrial emissions.
• Align policies limiting air pollution with climate change, energy and other environmental policies at the EU, national and local levels to capitalize on co‐benefits. Policies should be targeted at different levels of governance.
• Improve spatial planning to reduce motorized traffic and provide accessible and safe infrastructure for active and greener travel.
• Develop and implement policies to discourage and phase out outdoor and indoor fossil and solid fuels for heating, cooking and recreational purposes, accompanied by awareness‐raising campaigns.
• Incentivize cleaner forms of energy for heating and cooking, which do not adversely affect indoor and outdoor air quality, such as heat pumps, solar power or geothermal energy.
• Support citizens to actively engage and participate in developing local plans to reduce emissions of air pollutants. Make sure information on outdoor air pollution at the local and national levels is easily available for the public.
• Protect sensitive populations and vulnerable groups from air pollution; for example, do not locate new schools or nursing homes next to busy roads. Where existing schools and other buildings with sensitive populations and vulnerable groups are situated next to busy roads, incentivize the use and correct maintenance of air filters and purifiers to avoid or decrease infiltration of outdoor air pollution.

© 2026 International Agency for Research on Cancer / WHO. Used with permission. References: • Directive (EU) 2024/2881 of 23 October 2024 on ambient air quality and cleaner air for Europe (recast). *OJEU*. 2024;**374**:1–45. Available from: https://eur-lex.europa.eu/eli/dir/2024/2881 [73]. • Directive 2010/75/EU of 24 November 2010 on industrial emissions (integrated pollution prevention and control). *OJEU*. 2010;**L334**:17–119. Available from: https://eur-lex.europa.eu/eli/dir/2010/75 [83]. • European Union Action Plan: Towards Zero Pollution for Air, Water and Soil. Brussels: European Commission; 2021. Available from: https://eur-lex.europa.eu/legal-content/EN/TXT/?uri=CELEX:52021DC0400 [79]. • Measures to reduce emissions of air pollutants and greenhouse gases: the potential for synergies. Copenhagen: European Environment Agency (EEA); 2022. Available from: https://www.eea.europa.eu/publications/measures-to-reduce-emissions-of [84]. • Global air quality guidelines: particulate matter (PM2.5 and PM10), ozone, nitrogen dioxide, sulphur dioxide and carbon monoxide. Geneva: World Health Organization; 2021. Available from: https://iris.who.int/handle/10665/345329 [12]. • Guidelines for indoor air quality: household fuel combustion. Geneva: World Health Organization; 2014. Available from:https://apps.who.int/iris/bitstream/handle/10665/141496/9789241548885_eng.pdf [82]. • Personal‐level actions to reduce air pollution exposure in the WHO European Region. Copenhagen: WHO Regional Office for Europe; 2024. Available from: https://iris.who.int/bitstream/handle/10665/375889/WHO-EURO-2024-9115-48887-72806-eng.pdf?sequence=1 [44].

### 3.1 Presentation of the recommendation for policymakers and key stakeholders

#### 3.1.1 For outdoor air pollution

*Fully align EU air quality limit values with the WHO global air quality guidelines for outdoor air pollution, without delay. At the local and national levels, develop and implement plans to ensure that all air pollutants comply with the WHO guidelines.*

In 2021, WHO developed new health‐based air quality guidelines for major air pollutants based on all nonaccidental mortality and cause‐specific respiratory mortality (Table 1). It is important to align the legally binding EU limit values with these guidelines. EU limit values are based on the principle that achieving the limit values should be technically and economically achievable, whereas the WHO guidelines only assess the health evidence. A health impact assessment conducted in the framework of the new EU directive [73], found that the benefits of reducing air pollution levels to these guidelines would far exceed the costs.

In 2024, the EU has decided upon new limit values to be met in 2030 [73]. While these new limit values are a major improvement compared with the previous limit values, they are still twice as high as the WHO guidelines (Table 1). Table 2 illustrates that about a 5% reduction in lung cancer incidence is possible when the WHO guidelines are achieved relative to the new EU limit values. Therefore, it is important for national, regional and local authorities to be more ambitious than the legally binding EU limit where feasible. Cities can also use the WHO guidelines to guide their air quality policies. In the framework of the WHO European Healthy Cities Network, sustainable policies have been developed. Further development of monitoring systems is useful.

*Ensure greening of heavy industry.*

Policies to reduce greenhouse gas emissions from large industries should be accompanied by policies to directly reduce emissions of pollutants that are damaging to health. To push for cleaner, more sustainable and energy‐efficient industries, as stated by the European Green Deal [74], it is important that governments provide subsidies to industries to reduce carbon dioxide emissions.

*No combustion for energy production at the EU level.*

In line with climate change mitigation, no burning of fossil fuels should be used for electricity production at the EU level, including a ban on coal‐fired power plants. Biomass combustion results in high particle emissions and, as such, is a nonsustainable energy source from the health point of view. Heating systems should not rely on burning oil, coal gas or wood [70].

*Align policies limiting air pollution with climate change, energy and other environmental policies at the EU, national, and local levels to capitalize on co‐benefits. Policies should be targeted at different levels of governance.*

Because burning fossil fuels is an important source of outdoor and indoor air pollution, policies aimed at reducing air pollution emissions would also be beneficial to reduce carbon dioxide emissions and contribute to addressing climate change. Co‐benefits of climate change and health policies have been identified previously [75, 76]. This requires actions on multiple levels, for example, at the EU level to set emission standards for motor vehicles, aviation and shipping, at the national level to promote major public transportation infrastructure, and at the city level to address local sources, including wood burning, and promote safe cycling and improve the pedestrian infrastructure [70]. Policies stimulating green infrastructure (trees) in cities reduce urban heat and air pollution in cities [77].

*Cities should provide accessible and safe infrastructure for active and greener travel.*

Active travel is a relatively easy and time‐efficient way for citizens to reduce emissions of air pollutants and also to provide advantages to individuals from increased physical activity, which is beneficial for both cancer prevention and survival from cancer [46, 47, 78], as well as for the prevention of other major causes of especially cardiometabolic and morbidity and mortality in the EU. To facilitate cycling and walking, a safe infrastructure needs to be developed in urban areas to avoid traffic accidents. Many cities in the EU have made progress in the past decade. Reducing traffic congestion is a co‐benefit of active travel policies [44, 46].

*Cities or municipalities should discourage the burning of materials.*

Because residential wood burning has become a larger source of outdoor air pollution, reducing the burning of wood is an important policy [60]. Policies may stimulate voluntary avoidance of burning through information campaigns, by subsidizing alternatives to wood burning or by prohibiting wood burning. A variety of financial assistance programmes have been established to help households replace older wood‐burning devices and to adopt other practices to reduce emissions [79].

*Support citizens to actively engage and participate in developing local plans to reduce emissions of air pollutants. Make sure information on outdoor air pollution at the local and national levels is easily available for the public.*

Engaging citizens in shared decision‐making, including in planning, will lead to better acceptance of initiatives to reduce air pollution, which have the potential to provoke opposition. Citizens are knowledgeable about their own neighbourhoods, and including the specific needs and considerations of the community has been recognized at the EU level in environmental impact assessment directives [80], which now include assessment of impacts on population and health. Well‐established tools for the involvement of citizens in decision‐making and the creation of trust are available (deliberative democracy) [81].

Providing up‐to‐date information about air quality levels for, or preferably homes, is important to empower citizens and raise their awareness. In recent years, local level information on air quality has been supplemented by citizens measuring their own pollution levels [63]. Citizen air monitoring is most useful when carried out in collaboration with the institutes responsible for monitoring pollution levels [63, 64].

#### 3.1.2 For indoor air pollution

Because a substantial part of indoor air pollution originates from the penetration of outdoor air pollutants, any policy measure targeted at outdoor air pollution (listed above) will also positively affect indoor air pollution.

*Develop and implement policies to discourage and phase out indoor burning of fossil and solid fuels for heating, cooking and recreational purposes, accompanied by awareness‐raising campaigns.*

Authorities are generally reluctant to prohibit activities in the private home environment. Awareness‐raising campaigns can encourage citizens to reduce indoor burning. In new housing, alternative heating and cooking facilities can be incorporated more easily, for example by connecting homes to communal warm water systems that use industrial waste heat. To avoid opposition to such policies, the energy costs should not exceed those of traditional energy systems [82].

*Incentivize cleaner forms of energy for heating and cooking, which do not adversely affect indoor and outdoor air quality, such as heat pumps, solar power or geothermal energy.*

Subsidizing the installation of nonfossil fuel heating systems may encourage their adoption. In several EU Member States, solar panels have been successfully installed on large numbers of homes [79, 82].

*For schools and other public buildings for which it is not possible to avoid penetration of outdoor air pollution, incentivize the use of air filters and purifiers. Buildings that are used by sensitive population groups, including children and older people (schools, homes for older people), should not be built near major roads.*

For existing schools that are near major roads, exposure to air pollution could be reduced by using air cleaners, such as proper filtration systems or mobile air cleaners. City subsidies for the schools would encourage this. A discussion on air filters and cleaners is provided by WHO [44]. The use of well‐functioning ventilation systems, which should be maintained correctly, is also encouraged, since air quality in classrooms is often of low quality. This measure has substantial co‐benefits in terms of respiratory diseases, including transmission of infection, and may contribute to better cognitive performance [78].

## 4 Conclusions

Air pollution remains the leading environmental risk factor for health in the EU, with the majority of the population living in areas where outdoor air pollution exceeds the 2021 WHO ambient air quality guidelines. Outdoor air pollution, particularly fine particulate matter, is a well‐established cause of lung cancer and may also contribute to the development of other cancer types. In 2023 alone, ambient particulate matter was estimated to be responsible for 28 000 cancer deaths across Europe. For the first time, the European Code Against Cancer, 5th edition (ECAC5), provides evidence‐based recommendations for both individuals and policymakers to address the cancer burden associated with citizens' exposure to outdoor and indoor air pollution.

Effectively reducing air pollution requires coordinated action at all levels: local, national and EU‐wide‐involving authorities, major industries and citizens. A crucial step is to align EU air quality standards with the more stringent 2021 WHO guidelines. This should be supported by comprehensive policy measures, including stricter regulation of combustion emissions, the promotion of active, safe and sustainable transportation, and integration of air quality policies with climate change mitigation strategies.

Individuals also play an important role. Citizens can reduce their contribution to air pollution and protect themselves from cancer by minimizing activities that involve combustion, such as driving cars or burning wood and coal, both indoors and outdoors. To further protect their health, individuals can avoid pollution hotspots and limit walking or cycling along routes with heavy traffic, especially during periods of high pollution. Additionally, public advocacy for stronger environmental policies, such as urging authorities to implement stricter regulations and cleaner urban transport, can drive meaningful change.

In summary, addressing the cancer burden from air pollution in the EU requires a combination of robust policy action and informed individual choices. By working together to reduce emissions and exposure, significant progress can be made towards cleaner air and improved public health.

## Conflict of interest

The authors GH, MvT, MR, SHJJ, NV, MA, IB, QC, BF, RG, DC, AF, HZ, JS, EDS, DR, CE, HK declare no conflicts of interest. Where authors are identified as personnel of the International Agency for Research on Cancer/World Health Organization, the authors alone are responsible for the views expressed in this article and they do not necessarily represent the decisions, policy or views of the International Agency for Research on Cancer/World Health Organization.

## Author contributions

GH and MvT were responsible for writing the first version of the manuscript. All authors gave critical revisions on the intellectual content of the manuscript and approved the final manuscript.

## Supporting information


**Annex S1**. European Code Against Cancer, 5th edition. © 2026 International Agency for Research on Cancer / WHO. Used with permission.

## Data Availability

The data that support the findings of this study are available in Figs 1 and 2 and Tables 1, 2, 3 and the Supporting Information of this article.
